# Global Gene Expression Analysis of the Brainstem in EV71- and CVA16-Infected Gerbils

**DOI:** 10.3390/v12010046

**Published:** 2019-12-30

**Authors:** Yi-Sheng Sun, Zhang-Nv Yang, Fang Xu, Chen Chen, Hang-Jing Lu, Jian-Min Jiang, Yan-Jun Zhang, Han-Ping Zhu, Ping-Ping Yao

**Affiliations:** Key Lab of Vaccine, Prevention and Control of Infectious Disease of Zhejiang Province, Zhejiang Provincial Center for Disease Control and Prevention, Hangzhou 310015, China; yshsun@cdc.zj.cn (Y.-S.S.); znyang@cdc.zj.cn (Z.-N.Y.); fxu@cdc.zj.cn (F.X.); cchen@cdc.zj.cn (C.C.); hjlu@cdc.zj.cn (H.-J.L.); jmjiang@cdc.zj.cn (J.-M.J.); yjzhang@cdc.zj.cn (Y.-J.Z.)

**Keywords:** enterovirus 71, coxsackievirus 16, gerbils, neuropathogenesis, cytokine dysregulation

## Abstract

Enterovirus 71 (EV71) and coxsackievirus A16 (CVA16) are the two most important pathogens of hand, foot, and mouth disease (HFMD). However, the neuropathogenesis of EV71 and CVA16 has not been elucidated. In our previous study, we established gerbils as a useful model for both EV71 and CVA16 infection. In this work, we used RNA-seq technology to analyze the global gene expression of the brainstem of EV71- and CVA16-infected gerbils. We found that 3434 genes were upregulated while 916 genes were downregulated in EV71-infected gerbils. In CVA16-infected gerbils, 1039 genes were upregulated, and 299 genes were downregulated. We also found significant dysregulation of cytokines, such as IP-10 and CXCL9, in the brainstem of gerbils. The expression levels of 10 of the most upregulated genes were confirmed by real-time RT-PCR, and the upregulated tendency of most genes was in accordance with the differential gene expression (DGE) results. Our work provided global gene expression analysis of virus-infected gerbils and laid a solid foundation for elucidating the neuropathogenesis mechanisms of EV71 and CVA16.

## 1. Introduction

Hand, foot, and mouth disease (HFMD) is a common infectious disease in children under 5 years old. HFMD courses are usually mild or self-limited, but a few patients could have severe symptoms or even die. In recent years, there have been several outbreaks in the West Pacific region, such as in China, Japan, Thailand, and so on [1,2,3]. There are also many HFMD case reports in Europe, such as in France and Denmark [4,5]. Obviously, HFMD is becoming an increasing public health problem worldwide. More than 20 enteroviruses can lead to HFMD. Among them, enterovirus 71 (EV71) and coxsackievirus A16 (CVA16) are the two most important pathogens. Over 90% of severe and fatal infections are caused by enterovirus 71. In China, a total of 13,686,985 HFMD cases were laboratory confirmed from 2008 to 2015. Approximately 43.6% of patients were infected by EV71, and 24.9% of patients were infected by CVA16 [6]. The EV71 virus can infect the central nervous system (CNS), and EV71 virus infection can lead to meningitis, encephalitis, encephalomyelitis, or acute flaccid paralysis [7]. The CVA16 virus can also breach the blood–brain barrier and damage the CNS. If an HFMD patient’s CNS becomes infected, they usually have severe symptoms or even die [8]. Though the disease can be ultimately cured, the patients may have several sequelae. To date, the neuropathogenesis of EV71 and CVA16 has not been elucidated.

Humans are the natural hosts of EV71 and CVA16. There are not many ideal animal models for EV71 and CVA16 infection so far. Mouse and rhesus macaque models have recently been established for both EV71 and CVA16 infection [9,10]. However, those two animal models have limitations. The mouse model is not quite sensitive to enteroviruses. Mice older than 7 days do not develop pathological changes. There have been no reports of CNS lesions when rhesus macaques were infected by CVA16. The gerbil has been a suitable animal model for medical use for more than 40 years, especially for cerebral infarction. Our team established a gerbil animal model for infection with both the EV71 and CVA16 viruses successfully [2,3]. The gerbil model is more sensitive to the EV71 and CVA16 viruses than the mouse model. Gerbils that are 21 days old can still develop neurological disorders, such as focal shrinking of neurons and neuronophagia, in the brainstem after infection. In this study, we used a 100× the 50% lethal dose (LD_50_) of EV71 or CVA16 to separately infect gerbils. We extracted total RNA from the brainstem and used RNA-seq technology to analyze global gene expression. We found significant cytokine dysregulation in the brainstem in EV71- and CVA16-infected gerbils and used RT-PCR to verify expression changes in 10 of the most upregulated genes. Our results might provide a valuable reference for clarifying the neuropathogenesis of EV71 and CVA16.

## 2. Materials and Methods

### 2.1. Viral Isolation and Incubation

The CVA16 clinical strain CVA16-196 (GenBank ID: KU580041), which belongs to genotype B1, was isolated from swab samples from a 37-month-old HFMD patient in Hangzhou in 2008. The EV71 clinical strain 58,301 (GenBank ID: KX588236) is grouped into genotype C4 and was isolated from swab samples from a 12-month-old boy in 2008. Vero cells were cultured in modified Eagle’s medium (MEM) supplemented with 10% fetal bovine serum, 100 units/mL penicillin, and 100 μg/mL streptomycin at 37 °C in 5% CO_2_. The EV71 and CVA16 viruses were propagated in Vero cells, and the virus stocks were collected when a cytopathogenic effect was observed. The titers of EV71 and CVA16 were determined using previously described standard methods [3].

### 2.2. Gerbil Infection Experiments

Gerbils were purchased from the Animal Center of the Zhejiang Academy of Medical Sciences, Hangzhou, China. Gerbils that were 21 days old were inoculated separately with 1 × 10^4.6^ (the 50% tissue culture infectious dose (TCID_50_)) EV71 or 3.2 × 10^3.0^ CVA16 viruses via the intraperitoneal (IP) route. At 4 days post-infection, gerbils were anesthetized by ether, and the brainstems were collected. Phosphate buffer saline (PBS) was used as a negative control. The animal experimental protocols were approved by the animal ethics committee of Zhejiang Academy of Medical Sciences with the project number of 2019-053 (11 March 2019). The methods were carried out in accordance with the guidelines of the Administration of Affairs Concerning Experimental Animals of the People’s Republic of China and the principles of the Declaration of Helsinki.

### 2.3. Next-Generation Sequencing of mRNA and Data Processing

For transcriptome sequencing, total RNA was extracted from 25 mg of brainstem from each gerbil by using an RNeasy Mini Kit (Qiagen, Valencia, CA, USA). To minimize individual differences, three experimental and control samples were mixed and regarded as one sample for each group. Each group had two replicates. The RNA concentrations were measured using a Qubit RNA Assay Kit (Life Technology, Rockville, Maryland, USA), and the RNA quality was detected by an Agilent Bioanalyzer 2100 (Agilent Technologies, Santa Clara, CA, USA). Sequencing libraries were generated by an NEB Next Ultra RNA Library Prep Kit (NEB, Ipswich, MA, USA) and sequenced by the Illumina HiSeq-PE150 platform. Clean data for de novo assembly were generated by removing adapter reads, reads that contained more than 10% unknown bases, and low-quality sequences. Transcriptome assembly was performed using Trinity software (v2.4.0) with min_kmer_cov set to 2 by default and all other parameters set to default. Over 80% of the total reads in each sample were mapped to the assembled transcriptomes (Tables S1 and S2).

All the clean data from the EV71 and CVA16 groups were deposited in the NCBI GEO database (accession number GSE123550 for EV71 and GSE121971 for CVA16). The function of assembled unigenes was annotated based on Nr (NCBI not-redundant protein sequences), Pfam (protein family), COG (Clusters of Orthologous Groups of proteins), Swiss-Prot, and KO (Kyoto Encyclopedia of Genes and Genomes (KEGG) Ortholog) databases. Gene expression levels were estimated by RSEM software (v1.2.15), and differential gene expression (DGE) analysis was performed using the DESeq R package (1.10.1). The adjusted *p*-values were used to control the false discovery rate by the Benjamini and Hochberg’s method. The cut-off value for assignment of the significant differentially expressed genes (DEGs) in this study was padj < 0.05, and |Log2fold change|>1. Gene ontology (GO) enrichment analysis of the DEGs was implemented by the GOseq R package (1.10.0) [11], which can adjust for gene length bias in DEGs. Statistical enrichment of DEGs in KEGG pathways was analyzed using KOBAS software (v2.0.12).

### 2.4. Quantitative RT-PCR (qRT-PCR) Validation

Total RNA was extracted from the brainstem of individual gerbils, and the RNA was converted to cDNA by a PrimeScript II 1st Strand cDNA Synthesis Kit (Takara, Kyoto, Japan). Quantitative RT-PCR was performed by SYBR Premix Ex Taq II (Takara) on an Applied Biosystems instrument. The experiment was repeated twice. The primer sequences are shown in Table 1 below and Table S3. β-actin was used as an internal control.

## 3. Results

### 3.1. Different Gene Expressions in the Brainstem of Gerbils Infected by EV71 or CVA16

**Figure 1:**
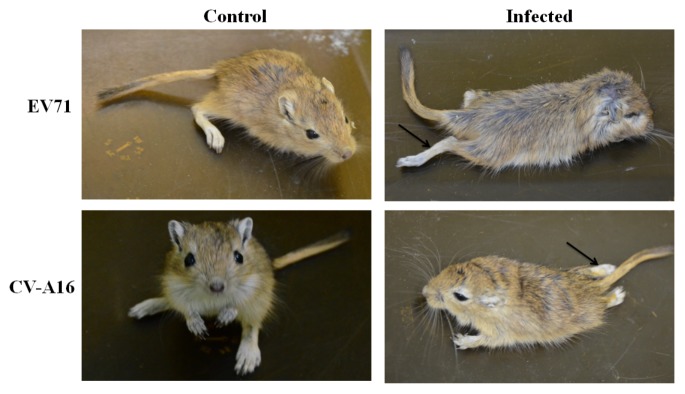
EV71- and CVA16-infected gerbils. At 21 days old, gerbils were infected with 100× LD_50_ of EV71 virus (1 × 10^4.6^ TCID_50_) and CVA16 virus (3.2 × 10^3.0^ TCID_50_). PBS was used as a negative control. Four days post-infection, pictures were taken, and the arrows indicate the limb paralysis of infected gerbils.

**Figure 2:**
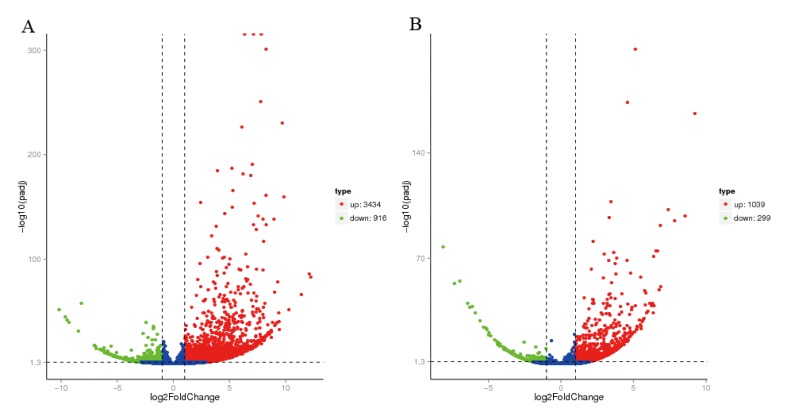
A volcano plot showing DGE in the brainstem of gerbils infected by EV71 (**A**) and CVA16 (**B**) compared to that of the control gerbils. The blue dots mean not significant changed genes. The horizontal dotted line represents the threshold of statistical significance, and the vertical dotted line represents the threshold of fold change.

We used EV71 at a TCID_50_ of 1 × 10^4.6^ and CVA16 at a TCID_50_ of 3.2 × 10^3.0^, which are both 100 times the LD_50_, to infect 21-day-old gerbils. At 4 days post-infection, both the EV71- and CVA16-infected gerbils developed slow movement or even hind limb paralysis (Figure 1). We isolated the brainstems from gerbils and extracted total RNA. DGE analysis showed that 3434 genes were upregulated while 916 genes were downregulated in EV71-infected gerbils compared with those in the control gerbils (Figure 2). In CVA16-infected gerbils, 1039 genes were upregulated and 299 genes were downregulated.

### 3.2. Target Gene Prediction and Functional Analysis

**Figure 3:**
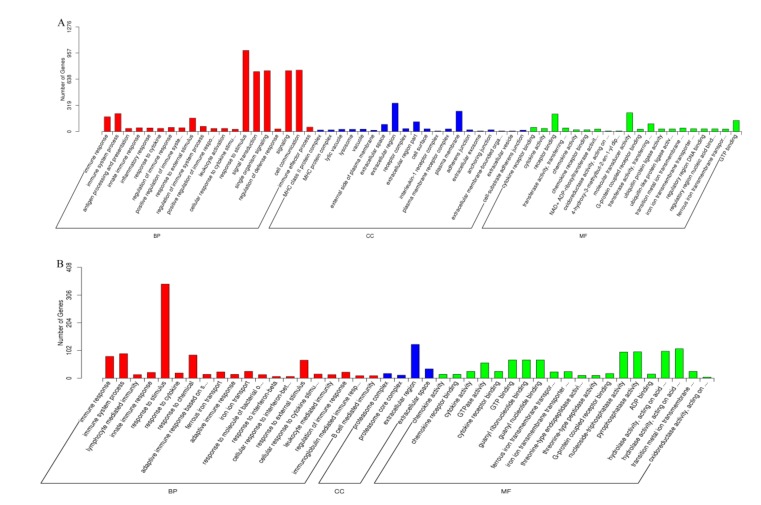
GO enrichment chart for the DEGs in the EV71-treated and CVA16-treated groups. DEGs were classified into three types: Biological process (BP), cellular component (CC), and molecular function (MF). (**A**) EV71-treated group compared to the control group. (**B**) CVA16-treated group compared to the control group.

GO term enrichment highlights the differential expression of genes from the genome background. GO terms are divided into three types: Biological process, cellular component, and molecular function. As shown in Figure 3A, GO enrichment showed that a total of 3189 genes were differentially expressed in the EV71-treated group compared with those in the control group. Response to stimulus (988 genes) and signal transduction (730 genes) were the top two biological process categories. The top two molecular function categories were reporter binding and molecular transducer activity, while extracellular region and plasma membrane were the top two cellular component categories. As shown in Figure 3B, GO enrichment also revealed 1019 DEGs in the CVA16-infected group compared with those in the control group. The top biological process and cellular component categories in the CVA16-infected group were the same as those in the EV71-infected group. However, the top two molecular function categories in the CVA16-infected group were pyrophosphatase activity and nucleoside-triphosphatase activity, which were different from those in the EV71-infected group.

### 3.3. KEGG Pathway Scatter Plot

**Figure 4:**
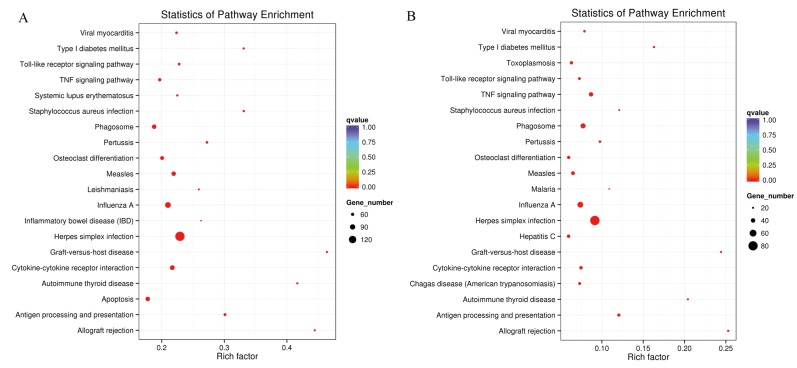
The top 20 enriched KEGG pathways in the EV71-infected group (**A**) and CVA16-treated group (**B**).

KEGG pathway analysis is applied to enrich DEGs in related biological, metabolic, and signaling pathways. The top 20 enriched KEGG pathways are shown in Figure 4. The top five most significantly enriched pathways in the EV71-infected group were herpes simplex infection, allograft rejection, graft-versus-host disease, antigen processing and presentation, and autoimmune thyroid disease. In the CVA16-infected group, the top five most significantly enriched pathways were herpes simplex infection, graft-versus-host disease, allograft rejection, autoimmune thyroid disease, and influenza A. Herpes simplex infection was the most significantly enriched pathway in both the EV71-infected and CVA16-infected groups, with 148 and 81 related genes, respectively.

### 3.4. Gene Expression Validation by RT-PCR

**Figure 5:**
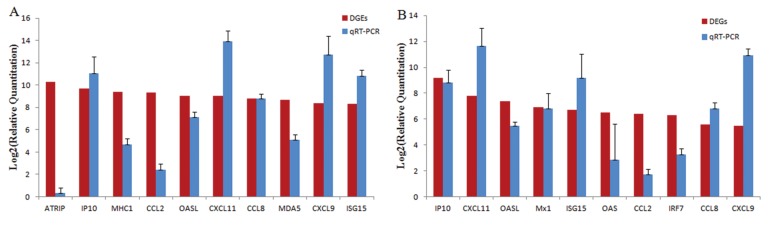
Confirmation of the most upregulated genes by qRT-PCR. (**A**) In the EV71-infected group, 10 of the most upregulated genes were selected and analyzed: ATRIP, IP10, MHC1, CCL2, OASL, CXCL11, CCL8, MDA5, CXCL9, and ISG15. (**B**) In the CVA16-infected group, IP10, CXCL11, OASL, Mx1, ISG15, OAS, CCL2, IRF7, CCL8, and CXCL9 were selected and analyzed.

To confirm the DGE analysis results, we selected 10 of the most upregulated genes and used RT-PCR to detect expression changes. In the EV71-infected group, ATRIP, IP10, MHC1, CCL2, OASL, CXCL11, CCL8, MDA5, CXCL9, and ISG15 were selected and analyzed. The expression levels of these genes in the EV71-infected group were increased by 2^0.3^, 2^11.1^, 2^4.7^, 2^2.4^, 2^7.1^, 2^13.9^, 2^8.8^, 2^5.1^, 2^12.7^, and 2^10.8^, respectively, compared to those in the control group (Figure 5). In the CVA16-infected group, IP10, CXCL11, OASL, Mx1, ISG15, OAS, CCL2, IRF7, CCL8, and CXCL9 were selected, and the gene expression levels were increased by 2^8.8^, 2^11.7^, 2^5.5^, 2^6.8^, 2^9.2^, 2^2.9^, 2^1.7^, 2^3.3^, 2^6.8^, and 2^11.0^, respectively. Although the gene expression levels were not the same as those indicated in the DGE results, the upregulation tendency of most genes was in accord with the DGE results and the results of a high dose (1000× LD_50_) of virus-infected groups (Figure S4).

## 4. Discussion

HFMD is a worldwide disease, especially for children under 5 years old. If HFMD pathogens, such as EV71 and CVA16, infect the CNS, the patients can become seriously ill. The course of disease progresses rapidly, leading to difficult treatment and poor prognosis. Although many neural cells, such as SK-N-SH, U251, and NSC-34 cells, have been used to study the pathogenesis of EV71 in vitro, few studies have elucidated the mechanism of EV71 in vivo [7,12,13]. CVA16 receives much less attention than EV71, and the neuropathogenesis of CVA16 is still unknown. Gene expression and cell signaling pathways in vivo are quite different than those in vitro. Global microRNA expression analysis was performed in EV71- and CVA16-infected human bronchial epithelial cells in vitro and rhesus monkey peripheral blood mononuclear cells in vivo [14,15]. However, no global analysis has been performed in the CNS in vivo. RNA-seq is a powerful tool to investigate the transcriptome and has been widely used in host–virus interaction research. In this study, we used RNA-seq to first analyze global gene expression in EV71- and CVA16-infected brainstems in vivo. We found significant dysregulation of cytokines, such as IP-10 and CXCL9, in the brainstem of gerbils. Our results provided an improved understanding of the neuropathogenesis of EV71 and CVA16.

Animal models are useful tools to study the pathogenic mechanism of EV71 and CVA16. Through animal models, we can isolate CNS organs, such as brainstems from EV71- or CVA16-infected gerbils, and use RNA-seq technology to detect the global gene expression of the CNS in vivo. Our team successfully established a gerbil model for EV71 and CVA16 virus infection [2,3]. Neuronal degeneration and neuronophagia, which mimic the clinical symptoms of HFMD patients, were detected in the brainstem and spinal cord of the gerbil model. The virus titers of the brainstem in the gerbil model were higher than those in other organs, with a TCID_50_ of 10^7.9^ per gram. The brainstem and spinal cord, which belong to the CNS, were important virus target organs in the EV71- and CVA16-infected gerbil. Severely ill or dead HFMD patients also usually have a CNS infection caused by EV71 or CVA16. Therefore, the gerbil model could be a suitable animal model to study the neuropathogenesis of EV71 and CVA16.

Through the DGE analysis, we found more than 1000 different expression genes. There were also some reports about differential expression of so many genes after EV71 or CVA16 infection. Jin identified 1825 genes that were upregulated and 129 genes that were downregulated in CVA16-infected HEK293T cells compared with the CVA16-non-infected samples [16]. Zhang found more than 1000 genes that were up- or downregulated in the peripheral blood mononuclear cells of EV71-infected rhesus monkey infants compared with the EV71-non-infected groups [17]. Among all DEGs, the number of upregulated genes was 3 times greater than that of downregulated genes in both EV71- and CVA16-infected gerbils. It seemed that genes tended to be upregulated in EV71- and CVA16-infected gerbils. This phenomenon can also be found in other studies. In CVA16-infected HEK293T cells, 1825 genes were upregulated, while only 129 genes were downregulated compared to those in the control [16]. In EV71-infected RD cells, 36 miRNAs were upregulated, and 9 were downregulated [18]. Through GO term and KEGG pathway analysis, more upregulated genes than downregulated genes were enriched in the three functional types and KEGG pathways. The number of upregulated genes was greater than that of downregulated genes in each category (Figure S1). Genes that were upregulated after EV71 or CVA16 infection seemed to play more important roles than those that were downregulated.

Through the GO enrichment analysis, we found that response to stimulus was the top biological process category. A total of 767 of the 988 DEGs in response to stimulus were upregulated. Cytokines play an important role in the immune system in response to viral infection. Among the 10 most upregulated genes in EV71-infected gerbils, 6 were cytokines, including 5 chemokines and 1 interferon-stimulated gene (ISG). IP-10, known as C-X-C motif chemokine 10 (CXCL10), is a small cytokine that responds to IFN-γ and is one of the most upregulated genes in EV71- and CVA16-infected gerbils [19]. The expression level of IP-10 was found to be elevated significantly in EV71-infected patients, especially in severely ill patients with pulmonary edema. In our study, the expression levels of IP-10 were increased by 2^12.2^ and 2^9.5^ after infection with EV71 and CVA16, respectively. IP-10 can protect mice from various viral infections, such as coxsackievirus B3 and dengue virus [20]. The boost of IP-10 can reduce the viral burden and increase the survival rate in EV71-infected mice. IP-10 might be a crucial cytokine during the host–pathogen interaction. CXCL-9, another cytokine that was increased in patients with pulmonary edema [21], was also one of the most upregulated genes in EV71- and CVA16-infected gerbils. In the statistical analysis of the pathway enrichment, we also found that the cytokine-cytokine receptor interaction pathway was one of the top 20 enriched upregulated pathways in both the EV71- and CVA16-infected groups. In addition to the cytokines that were among the 10 most upregulated genes, we also used RT-PCR to detect the expression of other cytokines, such as CCL3, CCL5, CCL19, CXCL13, IL15, and IL1β (Figure S2). All the cytokines were upregulated, which was consistent with the RNA-seq results in EV71-infected gerbils. In CVA16-infected gerbils, although DGE analysis did not show any expression changes in CCL19, CXCL13, IL15, and IL1β, all six genes were found to be upregulated by the qRT-PCR detection. Our results indicated that there was a dysregulation of cytokines or even regard as a “cytokine storm” in the brainstem of EV71- and CVA16-infected gerbils.

Through KEGG pathway analysis, herpes simplex virus (HSV) was the most significantly enriched pathway. Herpes simplex infection was also the most upregulated enriched pathway in the EV71-infected and CVA16-infected groups (Figure S3). This result was interesting. Patients infected by herpes simplex virus could have clinical symptoms similar to those of HFMD patients, such as rash, fever, and oral vesicles [22]. Though EV71 and CVA16 are RNA viruses, while HSV is DNA virus, there are several similar immune responses after virus infection in the CNS. A robust level of IP-10 measured in the brains of HSV-infected mice was also found in the CNS of EV71-infected mice and patients [23,24,25]. Increased cytokine levels, such as those of IL1β and CCL5, in herpes simplex virus-infected patients were detected in HFMD patients. Occasionally, it is difficult to distinguish whether patients were infected by herpes simplex virus or an enterovirus based only on the clinical symptoms and epidemiological data. Laboratory detection results should be combined to make a judgment. The known neuropathogenesis mechanism of herpes simplex virus in humans could be a valuable reference to study that of EV71 and CVA16. The apoptosis pathway was found among the enriched KEGG pathways of the EV71-infected group. We previously demonstrated that EV71 could induce apoptosis in gerbil muscle cells [26]. It seemed that EV71 might also induce apoptosis in the CNS of gerbils.

In conclusion, we first analyzed the global gene expression of the CNS in EV71-infected and CVA16-infected gerbils. The results showed that more genes were upregulated than downregulated when gerbils were infected with the EV71 or CVA16 virus. Cytokine dysregulation was found in the brainstem of EV71- and CVA16-infected gerbils. Herpes simplex infection was the most upregulated enriched pathway in EV71-infected and CVA16-infected groups, and we could study the neuropathogenesis of EV71 and CVA16 by referring to the known pathogenesis mechanism of herpes simplex virus. We also found an apoptosis pathway enriched in EV71-infected gerbils. Our results provide a solid foundation for elucidating the neuropathogenesis mechanism of EV71 and CVA16, as well as for preventing HFMD.

## Figures and Tables

**Table 1:** Primers for the selected genes.

Gene	Sequences	
	Forward Primer (5′-3′)	Reverse Primer (5′-3′)
ATRIP	GATGCAGGACCTCAACGCAAT	CCCACCAGCAGAAGCAGACAG
IP10	CCTCAGCGTAGCTTCCAATAC	AAGTCAAGCCCAGGAACAAG
MHC1	CAGGGAGATGTCAGCAGGGTA	GGAAGTGGGAAAACAGCGAGT
CCL2	AGGTTCAAGGATGCCGAGTT	GTGTTGGCTAGGCCAGATTC
OASL	TGGAGTATCTGGCTCGGGTT	CGCTTTGAGTCGGCTATCTT
CXCL11	CAAGCACGCCTTATACGACA	TTCCGGTTGCCAGTCACTTT
CCL8	TGAGAAGTGGGTCCAGTCATA	TCGTGGGTCAAGTTAGCATT
MDA5	GCAGGTCGGTGAGTGTGGGT	GGGTGGGGGCAGATGTTTGT
CXCL9	CCAGATTCGGCAAATGTGAA	CAGTGAAGGCATTCCGCTAA
ISG15	GGTCTTCTTGTACTTGCTCCTTCT	ACAGCGTCACCCTTATTAGCC
MxA-1	AAGGCGAAGACCTCTATTGC	GATGATTAAAGGGATGTGGC
OAS	TGGAGTATCTGGCTCGGGTTAA	ATCGCTTTGAGTCGGCTATCTT
IRF7	TGGCTTTATGGTTCAGTTTGTGA	CCAAGGCTCTGACTGGGAAG
β-actin	AACACCCCAGCCATGTACGTA	TCTCCGGAGTCCATCACAATG

## Supplementary Materials

The following are available online at https://www.mdpi.com/1999-4915/12/1/46/s1.
