# Supplementary material for: Global Gene Expression Analysis of the Brainstem in EV71- and CVA16-Infected Gerbils
Source: Viruses. 2019 Dec 30;12(1):46. doi: 10.3390/v12010046 (PMC7019476; doi:10.3390/v12010046)
Supplement: Supplementary file 1 [file viruses-12-00046-s001.pdf]

Global gene expression analysis of the brainstem in EV71- and CVA16-infected gerbils

Yi-Sheng Sun[1], Zhang-Nv Yang[1], Fang Xu[1], Chen Chen[1], Hang-Jing Lu[1], Jian-min Jiang[1], Yan-Jun Zhang[1], Han-Ping Zhu[1*], Ping-Ping Yao[1*]

1 Key Lab of Vaccine, Prevention and Control of Infectious Disease of Zhejiang Province, Zhejiang Provincial Center for Disease Control and Prevention, Hangzhou 310015, China; yshsun@cdc.zj.cn

*Correspondence: hanpingzhu@aliyun.com or pingpingyao@aliyun.com

Table 1. Reads mapping of the EV71 group.

Sample name	Total reads	Total mapped
**mC1**	43725700	37547818(85.87%)
**mC2**	41889070	35806060(85.48%)
**mC3**	41735672	35649268(85.42%)
**mT1**	40051872	34570358(86.31%)
**mT2**	38589668	32665406(84.65%)
**mT3**	37797808	32568650(86.17%)

mC1, mC2, mC3 are control samples. mT1, mT2, mT3 are experimental samples.

Table S2. Reads mapping of the CVA16 group

Sample name	Total reads	Total mapped
**Con_1**	31310904	25602538(81.77%)
**Con_2**	39529314	31793434(80.43%)
**Con_3**	26681092	21768076(81.59%)
**Y_1**	26740016	22124448(82.74%)
**Y_2**	37063880	30316226(81.79%)
**Y_3**	28037016	23107388(82.42%)

Con_1, Con_2, Con_3 are control samples. Y_1, Y_2, Y_3 are experimental samples.

Table S3. Primers for the selected genes

Gene	Sequences	
	Forward primer (5'-3')	Reverse primer (5'-3')
CCL3	GGTTAAGAATCCCTGGGTGTA	CTGATCTGGAGCTGAATGCC
CCL5	TGTATTCTCGAACCCACTTCTTC	TGCTGCTTTACCTACCGCTCC
CCL19	CCTCTTCTGGTCCTTGGTTTCTT	GCCTTTCGCTACCTCCTTATCC
CXCL13	TTCACTGGAGCGACGGGAGT	AGGCATCATAGAGCGGGTTC
IL15	CAGAGCCAGAGTTGGGAGGG	TGGAGGTACTGAATGGGAGATG
IL1β	CATTAGGAGCCCGGCCTTTAC	CAGCACCTCCCAAGCAGAACA

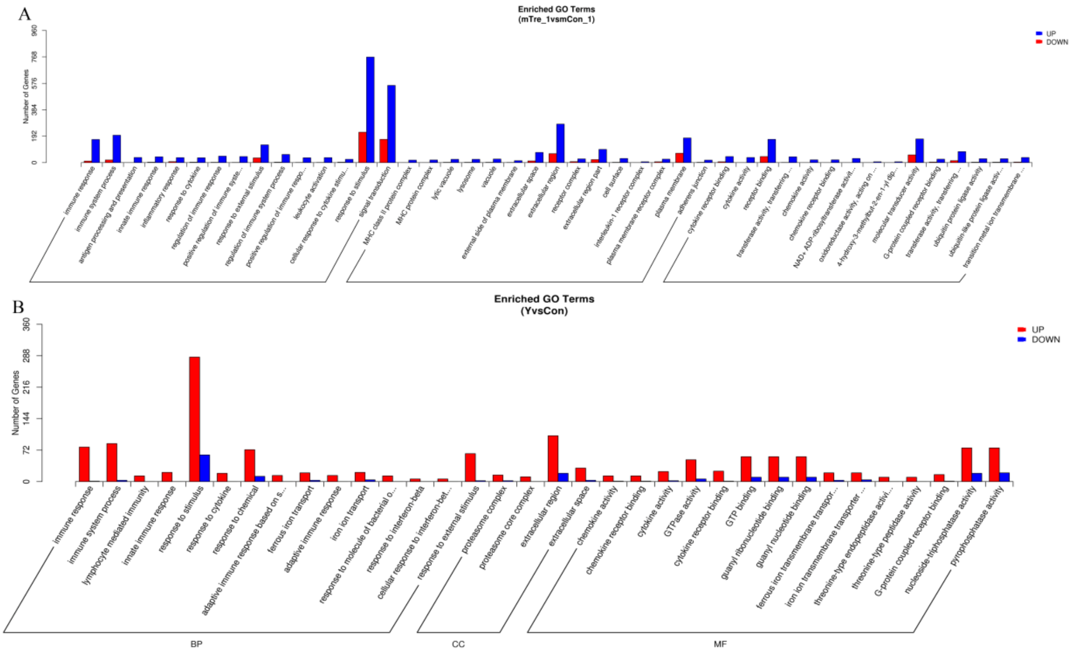

Figure S1. GO enrichment chart for the different expression genes in the EV71-treated and CVA16-treated groups. Both up-regulated and down-regulated genes were classified into three types: biological process, cellular component and molecular function. (**A**) EV71-treated group compared to the control. (**B**) CVA16-treated group compared to the control.

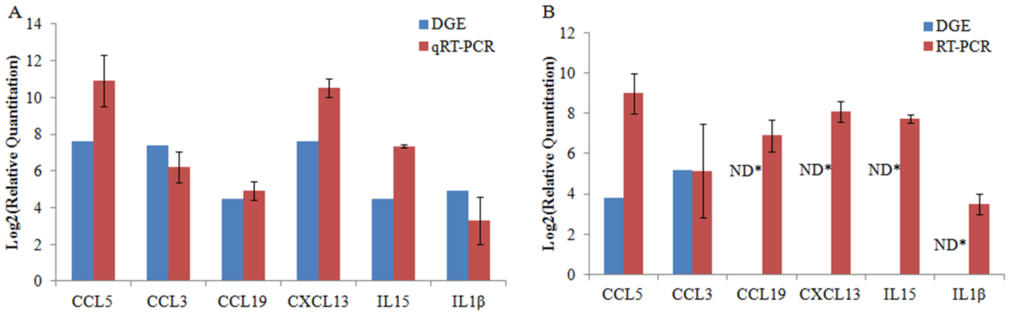

Figure S2. The confirmation of the selected genes by qRT-PCR. (**A**) In EV71- and (**B**) CVA16-infected group, CCL3, CCL5, CCL19, CXCL13, IL15 and IL1β were selected and analyzed. ND*: no data.

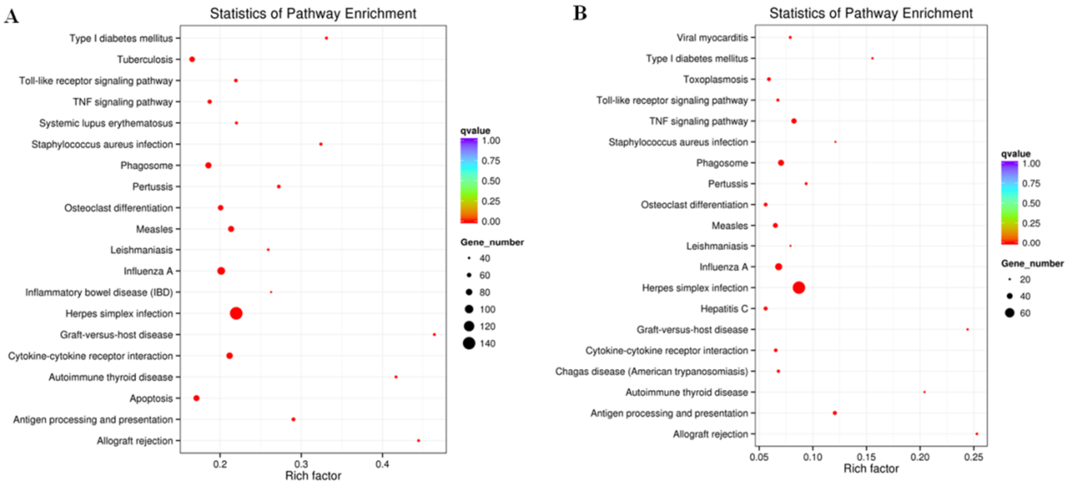

Figure S3. The top 20 enriched up-regulated KEGG pathways in EV71-infected group (**A**) and CVA16-treated group (**B**).

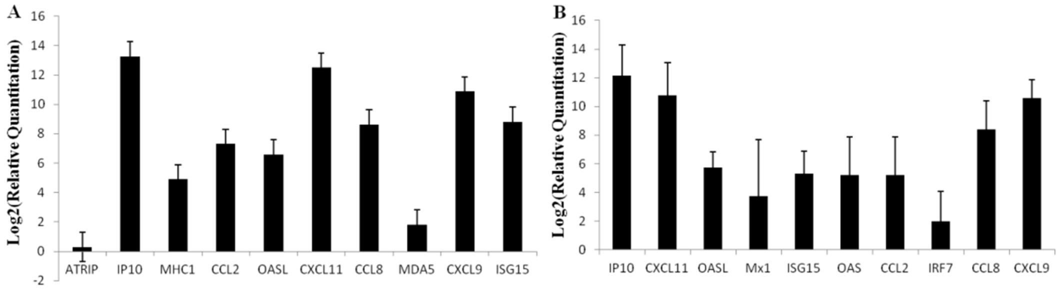

Figure S4. Confirmation of the most upregulated genes by qRT-PCR in 1000* LD_{50} of virus-infected gerbils. (**A**) In the EV71-infected group, ten of the most upregulated genes were selected and analyzed: ATRIP, IP10, MHC1, CCL2, OASL, CXCL11, CCL8, MDA5, CXCL9 and ISG15. (**B**) In the CVA16-infected group, IP10, CXCL11, OASL, Mx1, ISG15, OAS, CCL2, IRF7, CCL8 and CXCL9 were selected and analyzed.
